# The role of environmental technologies, institutional quality, and globalization on environmental sustainability in European Union countries: new evidence from advanced panel data estimations

**DOI:** 10.1007/s11356-024-31860-x

**Published:** 2024-01-10

**Authors:** Mucahit Aydin, Yasin Sogut, Azad Erdem

**Affiliations:** 1 Faculty of Political Sciences, Department of Econometrics, Sakarya University, Esentepe Campus, Serdivan/Sakarya, Turkey; 2 UNEC Research Methods Application Center, Azerbaijan State University of Economics (UNEC), Istiqlaliyyat Str. 6, Baku, Azerbaijan; 3 Faculty of Political Sciences, Department of Public Finance, Sakarya University, Esentepe Campus, Serdivan/Sakarya, Turkey

**Keywords:** Institutional quality, Load capacity curve (LCC), Globalization, Environmental sustainability, Panel, SDGs

## Abstract

Factors such as investments in environmentally clean technologies, globalization, and institutional quality significantly increase environmental quality. The study aims to provide light on how environmental technologies, institutional quality, globalization, and economic growth affect a sustainable environment. In addition, this study evaluates the European Union’s carbon zero target by 2050 and the results of achieving carbon neutrality by 2030, which was put on the agenda at the UN Climate Change Conference of the Parties (COP-26). For this purpose, ten countries (Germany, Austria, Denmark, Finland, France, Netherlands, Spain, Italy, Sweden, and Switzerland) that invest in the highest environmental technology in the European Union were selected in the study. The data range of the study is from 1990 to 2019. Also, the validity of the load capacity curve (LCC) hypothesis was investigated in these countries. The CCEMG and DCCE estimators were used to estimate long-run coefficients. When the panel was assessed as a whole, the LCC hypothesis was determined to be valid by both estimators. According to country-based results, it has been determined that the LCC hypothesis is valid only for Spain. The study also includes the following observations. (i) Environmental technologies increase LCF for Austria, improving environmental quality. (ii) Globalization reduces LCF for Austria. (iii) Institutional quality variable decreases LCF for Austria and increases LCF for Germany and France. These findings suggest that to attain a sustainable environment in the future, policymakers should raise research and development budgets for environmental technology, enhance the standards of institutions, and take globalization into account.

**Graphical abstract:**

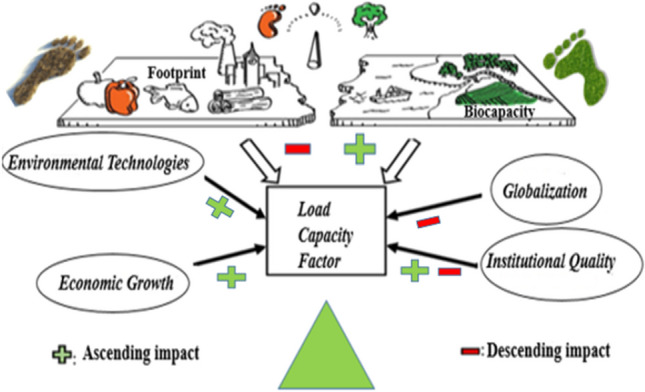

## Introduction

**Fig. 1:**
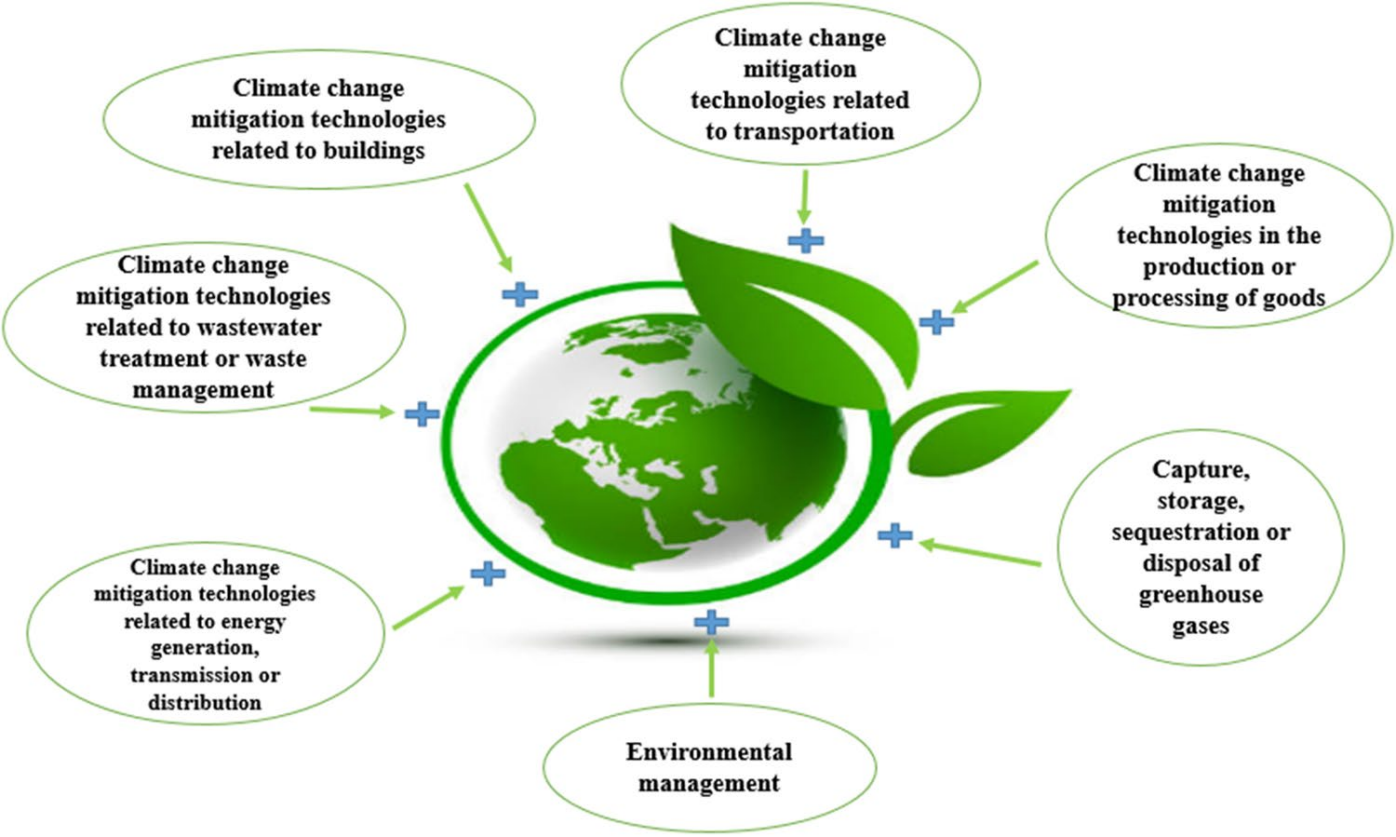
Content of environmental technologies. Source: compiled by authors from source OECD 2023)

Climate change and environmental degradation are critical topics on countries’ agendas today. The Twenty-six Conference of the Parties on Climate Change (COP26) has established guidelines for reducing environmental emissions for the Paris Agreement and the UN Framework Convention on Climate Change. In addition, the idea of minimizing environmental pollution is included in the 2030 Sustainable Development Goal (SDG)-7. Countries need to invest in environmental technologies to achieve these goals. These investments help increase the adoption of cleaner technologies globally (OECD 2023). Environmentally friendly technologies reduce the undesirable ecological consequences of practices, procedures, production equipment, and goods or services, such as creating new products (Klassen and Whybark 1999). The adoption of ecologically friendly technologies has increased, particularly in recent years, with widespread popular backing. Of course, this impacts environmental sustainability (Pata et al. 2023b). Environmental technologies developed mainly in building construction, and public transport activities increase environmental sustainability. Environmental technologies play a major role in preventing environmental pollution by capturing, storing, and disposing of greenhouse gases, energy generation, transmission, and distribution. Likewise, technological innovations and environmental management in wastewater treatment and waste management offer us a more livable environment (OECD 2023). Figure 1 shows the content of environmental technologies.

Two major environmental initiatives were implemented in 2019 with the European Green Deal, which has objectives nearly identical to those of the Paris Climate Agreement. The first environmental policy calls for increasing greenhouse gas emission reductions to at least 55% by 2030. Developing and diffusing environmental technology for the EU can be crucial in lowering greenhouse gas emissions. Another environmental objective is making the European continent the first climate-neutral zone by 2050. The proposed “Fit for 55” green package, which comprises several legislative measures, was submitted to the commission in 2021 to achieve the environmental goals that the EU had decided upon in this respect. This harmonizing package attempts to bring about the necessary change regarding social, economic, and environmental concerns and aligns the EU with its 55% objective. The budget for the EU’s fiscal years 2021 to 2027 was designed to aid the move toward climate neutrality (Sikora 2021; European Council (EC) 2023).

Globalization has also been connected to environmental pollution in addition to environmentally sensitive technology. Globalization has hastened the integration of countries into the global market. Moreover, globalization has impacted human existence regarding its social, economic, and environmental dimensions (Shahbaz et al. 2015; Aydin et al. 2023). Although it is difficult to give a satisfactory definition of globalization, it is generally defined as follows: It is expressed as facilitating the building of institutions at the international, national, regional, and local levels and the interaction of social, political, technological, commercial, economic, financial, and ecological processes (Rennen and Martens 2003). There are views on reducing or increasing the environmental pollution of globalization. Those who argue that globalization causes environmental pollution (Shahbaz et al. 2019) state that trade liberalization will increase with globalization, which will cause environmental pollution (Damania et al. 2003). In other words, while globalization promotes economic progress, it hastens the loss of natural resources in many emerging countries with low environmental regulations (Cole 2006; Copeland and Taylor 2004). Furthermore, growing ecological pressures brought on by globalization have resulted in changes to the ecosystem, environmental waste, a loss of biodiversity, and pollution (Panayotou 2000). On the other hand, those who argue that globalization improves environmental quality have stated that they will use more environmentally friendly technologies that do not cause environmental pollution as countries reach higher levels of economic growth (Stern 2004). This increases the environmental quality.

**Fig. 2:**
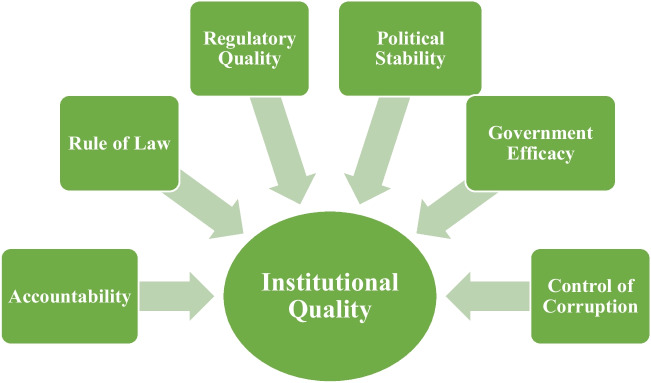
Institutional quality indicators. Source: compiled by authors from source Knoema Database, 2023

Along with globalization, the quality of institutions is also crucial in terms of environmental sustainability. Institutions are responsible for economic transactions and constitute laws and regulations that create social contracts to support or constrain organizational actions (North and Institutions 1990; Rothstein and Teorell 2008). Institutions determine the level of trade done to an economy. Boosting economic expansion can then have an impact on environmental degradation. Institutional quality includes six variables (political stability, regulatory quality, government efficacy, rule of law, accountability, and control of corruption) that help prevent environmental degradation. Moreover, it is predicted that environmental pollution will be higher in organizations that engage in bribery, rent-seeking, nepotism, and lobbying. Economies with weak institutions may face distinct environmental implications than those with robust institutions in this setting. Consequently, we must assess how institutional quality affects the environment to attain environmental sustainability (Amegavi et al. 2022). Figure 2 shows the institutional quality indicators.

A lot of research in the environmental literature (e.g., Dong et al. 2017; Wang et al. 2017; Hussain et al. 2022; Adebayo et al. 2022b; Hussain and Khan 2023; Zeng et al. 2023; Aydin and Bozatli 2022) employed carbon emissions as an ecological indicator. Degradation of the environment was measured using the ecological footprint in further research (Galli 2015; Solarin and Bello 2018; Kirikkaleli et al. 2021; Degirmencioglu Aydin and Aydin 2023; Aydin et al. 2023c). First used by Wackernagel and Rees (1998), ECOF only analyzes environmental degradation caused by human demand for natural resources. Several research studies have looked at the impact of environmental variables on ECOF, but none have looked at the supply side (biocapacity). The load capacity factor (LCF), developed by Siche et al. (2010), is a relatively new and widely used statistic. This is because the environment includes both the supply and demand sides (Pata and Isik 2021). In recent studies, the LCF has also been used as an ecological indicator (Fareed et al. 2021; Pata and Samour 2022; Awosusi et al. 2022a; Pata et al. 2023a; Pata et al. 2023b, Erdogan 2023; Erdogan 2024). LCF simultaneously assesses anthropogenic pressures on water, soil, and air and nature’s ability to respond to these pressures. LCF is calculated as biocapacity/ECOF and is an important and ideal indicator for assessing environmental sustainability. If this factor shows a value less than 1, it means that the environmental situation is unsustainable. A value greater than 1 indicates that the biocapacity is greater than ECOF. This means that natural resources can absorb human pressure and show ecological sustainability. LCF equal to 1 represents the environmental sustainability limit (Pata et al. 2023b). LCF is a more comprehensive instrument than carbon emissions and ECOF. Therefore, it provides a more complete and comprehensive contribution compared to previous environmental research (Awosusi et al. 2022a). In the LCF theory, the application of EKC works as an inverse mechanism according to carbon dioxide and ECOF. This is because LCF stands for ecological quality. The long-run revenue elasticity must be higher than the short-run to be valid (Pata et al. 2023a).

Economic growth is another critical environmental issue. Grossman and Krueger (1991) introduced the environmental Kuznets curve (EKC) to the literature, explaining an inverted U-shaped link between economic expansion and environmental improvement. The growth in gross national product up to the threshold amount increases environmental pollution in this curve. Environmental quality improves as income increases after reaching the threshold (Panayotou 1993). In other words, one of the causes for the growth in environmental quality after this threshold is that countries embrace environmentally friendly production technologies. Accordingly, the goal of government policy should be to shift the dynamics of economic growth in favor of green growth progressively (Aydin and Bozatli 2022). Besides the EKC hypothesis, the load capacity curve (LCC) hypothesis has recently been used as an environmental quality proxy. In this context, there is a U-shaped relationship between LCF and national income. In the first stages, as income increases, it leads to a decrease in environmental quality. Namely, it decreases the LCF. Thanks to economic growth, countries can turn to cleaner production technologies after a certain income. Environmental quality may be improved by raising environmental awareness. The LCC hypothesis describes the U-shaped link between income and LCF (Pata and Ertugrul 2023).

**Fig. 3:**
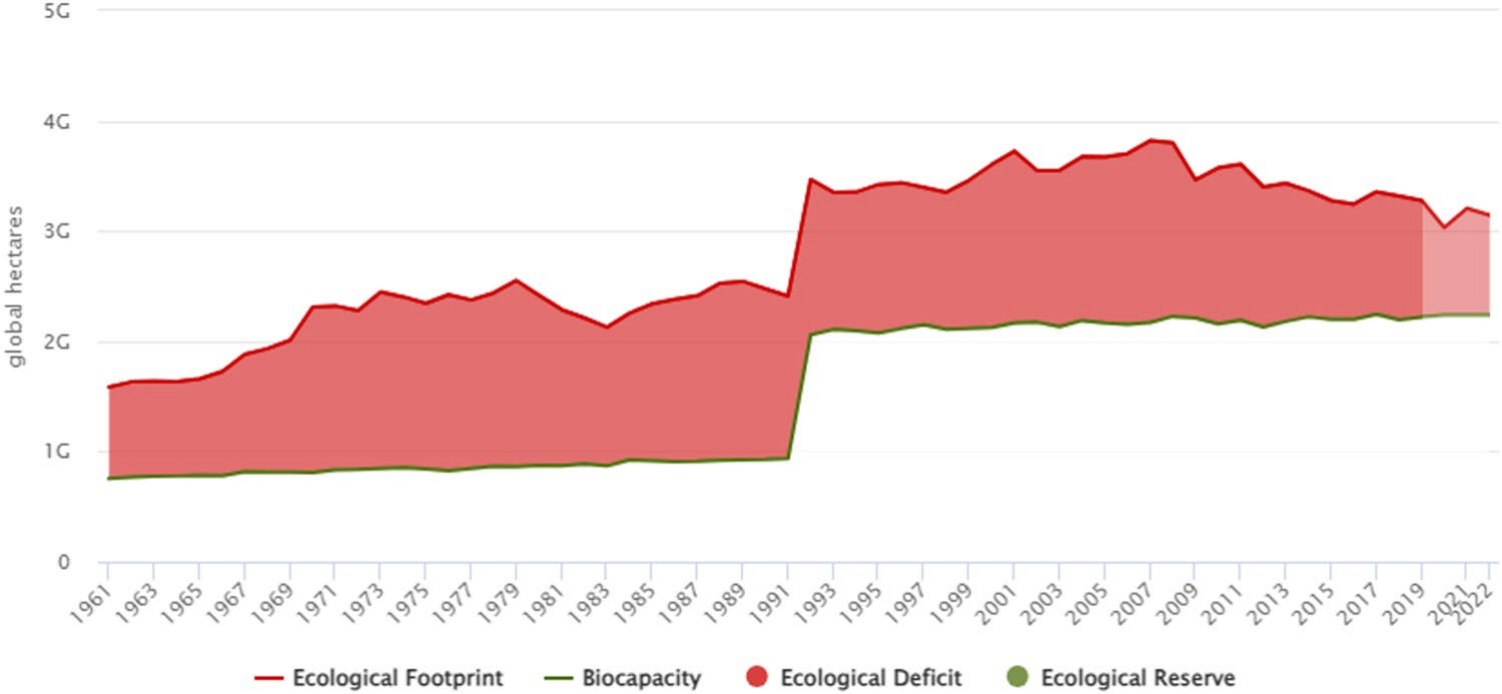
Trends in ecological footprint and biocapacity per capita in Europe between 1961 and 2022. Source: Global Footprint Network (2023)

Europe is one of the regions with an ecological deficit, meaning it consumes more than it has. It is seen that ECOF is above biocapacity in Europe in all the years in Fig. 3. This situation is undesirable for European countries in terms of environmental sustainability. Due to the lower-than-expected environmental quality in Europe, the investigation of the variables affecting the LCF stands out as an important research topic.

The ten countries investing in the highest environmental technology in the EU—Germany, Austria, Denmark, Finland, France, Netherlands, Spain, Italy, Sweden, and Switzerland—represent the country group of our study. The study investigates the connections between environmental technologies, globalization, institutional quality, and the load capacity factor for selected EU countries. The primary purpose of this study is to examine the role of environmentally friendly technologies in achieving sustainable environmental quality in European Union countries. Reducing environmental pollution is an important problem for many countries. To solve this problem, it is necessary to achieve the SDG-7 and SDG-9 goals. An LCF value greater than 1 contributes to the SDG targets for countries. Therefore, this study aims to achieve the SDG targets as well. It is seen that there is an intricate link between institutional quality, globalization, and environmental technologies for a sustainable environment. The first condition for the diffusion of environmental technologies across sectors is to ensure governance at the country level. In this context, institutional quality and environmental sustainability interact. The effects of these factors on environmental sustainability may vary depending on each other. In addition, the following points stated in the study are expected to contribute to the literature. (i) Adopting the load capacity factor as an indicator of environmental quality, the relationship between environmental technologies, globalization, institutional quality, and environmental degradation is the first study for selected EU countries. (ii) The amount of literature in which the load capacity factor has been examined within the framework of the LCC hypothesis is quite limited. (iii) How does the spread and development of environmentally friendly technologies impact the environment in European Union countries? The investigation explores how environmentally friendly technology affects long-term environmental quality as determined by LCF to provide a solution to this topic. The influence of institutional quality, economic growth, and globalization on LCF for the top ten EU countries investing in environmental technology is examined in this context. At this point, it is the first study to examine the effect of variables that interact and form a collective force for each other on environmental sustainability. (iv) International documents and declarations such as the United Nations Environment Programme, Paris Climate Agreement, UNFCCC, and Fit for 55 have imposed an obligation on the European Union countries to invest in environmental technologies. Considering all these obligations, this country group has been selected to obtain more efficient results. At the same time, other EU countries were not considered due to data limitations. By considering these countries, policy inferences were made for all EU countries. (v) Additionally, the new econometric approach utilized in the study produces reliable results, and (vi) this study results in fruitful policy recommendations for selected EU countries regarding the relationship between environmental technologies, globalization, institutional quality, and load capacity of economic growth.

This study is organized as follows. The second part introduces the literature. The third section describes the data and model. The fourth section presents the methodology and empirical results. The last part introduces the conclusion and policy recommendations.

## Literature review

The impact of environmental technologies, globalization, institutional quality, and economic growth on the LCF has been examined for Germany, Austria, Denmark, Finland, France, Netherlands, Spain, Italy, Sweden, and Switzerland, which have made the most investments in environmental technologies among EU countries. When the research in the literature on this issue is analyzed, it becomes clear that the factors provide various outcomes. Among the reasons for obtaining different results is that the method used in the test differs because the country group and data range are different. Studies related to the mentioned literature are as follows.

### Environmental technologies and LCF relationship

In the environmental literature, while CO2 emissions were used as an ecological indicator in previous studies, ECOF was used to measure environmental degradation in later studies. One of these studies is the study of Adebayo et al. (2022a). In this study, they examined the data for Portugal between 1980 and 2019. Innovative Morlet wavelet analysis reveals a new perspective on the link between technological innovation and carbon dioxide emissions. Morlet wavelet analysis shows that the technological innovation variable contributes positively to carbon emissions. Su et al. (2021) studied the link between the carbon dioxide emissions of the technological innovation variable using Bayer and Hanck cointegration, DOLS, and CCR causality tests in their study using quarterly data for Brazil between 1990 and 2018. According to the study’s findings, the variables have a long-term relationship. Furthermore, the results of DOLS and CCR show that increased technical innovation raises carbon dioxide emissions. In their study of the G7, Sharif et al. (2022) look at the contribution of green technology innovation to lowering carbon emissions. With data spanning 1995 to 2019, enhanced cross-section ARDL analysis was employed in the study. The results of the study show that green technology innovation harms carbon emissions. For big emerging market (BEM) nations, Destek and Manga (2021) seek to ascertain how technological innovation affects carbon emissions as well as ECOF. In this context, the effect of technological development on environmental degradation has been examined. For the abovementioned country, the data between 1995 and 2016 were analyzed using second-generation panel data. The study’s conclusions demonstrate that technological innovation successfully lowers carbon emissions. On ECOF, it has no appreciable impact. Therefore, a 1% increase in technological advances results in a 0.082–0.088% reduction in carbon emissions. Hussain and Dogan (2021) analysis of BRICS countries spans 1992–2016. This report recommends investing in environmental technology to lower ECOF. Ahmad et al. (2022) discovered similar results: environmental technologies minimize pollutants. Using data from 1990 to 2017, Akinsola et al. (2022) looked at the BRICS. The panel quantile regression model was used to conduct the study to determine the impact of technical innovation on ECOF. The findings show that technological innovation raises ECOF. In a study conducted in China between 1991 and 2017, Huo et al. (2023) discovered that environmental technologies increase ECOF.

In the latest investigations, LCF is used as an ecological indicator. Awosusi et al. (2022b) used data from 1980 to 2017 in their study of South Africa. In the study using the ARDL method, it has been proven that technological innovation improves environmental quality. Also, the analysis’s finding that the short-run coefficient value is less than the long-run elasticity supports the peripheral Kuznets curve theory. Additionally, both short-term and long-term LCF are predicted by technological advancement. Liu et al. (2022) used the ARDL bound test for Brazil with data from 1990 to 2018. They evaluated the impact of technological innovation on the LCF. According to the results of the study, it has been proven that there is a long-term interrelationship between the selected indicators. Technological innovations significantly improve ecological quality. At the same time, technological innovation gives rise to LCF, which suggests that it can predict environmental quality in the long run. Pata et al. (2023b) examine the effect of clean energy technologies on LCF in their study in the USA. The ARDL model was used in the study, which was conducted with data between 1974 and 2018. The results of the empirical study concluded that clean energy technologies do not affect LCF.

### Globalization and LCF relationship

Recently, as different globalization indices have been developed, the relationship between environmental pollution and globalization has become the focus again. The literature has two opposing viewpoints on the relationship between globalization and environmental contamination. The first of these claims is that globalization reduces pollution. In this regard, a study from 1990 to 2016 looked at the relationship between globalization and ECOF in 73 developing nations. According to this study, globalization reduces ECOF in Africa and South America. Furthermore, the environmental Kuznets curve validates this study’s African, Latin American, and Caribbean countries. That does not, however, apply to Asian countries (Jahanger et al. 2022). Akinsola et al. (2022) used the data covering 1990 and 2017 in their study of BRICS countries. As a result of the study, globalization significantly reduces ECOF. The advocates of the second view stated that globalization increases environmental pollution. In this view, Shahbaz et al. (2018) investigated the impact of globalization on CO2 emissions in Japan from 1971 to 2014. According to the findings, it was stated that globalization negatively affected the environment in Japan. Sharif et al. (2022) used cross-section augmented ARDL analysis with the data covering 1995–2019 for the G7 countries. Social globalization has proven to have a positive effect on carbon emissions. In addition to these two views, some studies show that the impact of globalization on environmental pollution is neutral. In this respect, the relationship between globalization and ECOF was examined for Malaysia in the period 1971–2014. In the study, Bayer and Hanck cointegration test was performed, and cointegration was determined. The results showed that globalization is not an essential determinant of ECOF (Ahmed et al. 2019).

Only some research includes globalization, LCF factors, and the association between globalization and carbon emission-ECOF. One of these recently reviewed studies is the study of Awosusi et al. (2022b). Their study examining South Africa used data from 1980 to 2017. In the analysis using the ARDL method, it has been proven that globalization increases LCF. In another study, Akadiri et al. (2022) used 1970–2017 data for India in their article study. The study explores the impact of financial globalization on LCF. Empirical results have proven a favorable relationship between financial globalization and LCF.

### Institutional quality and LCF relationship

The impact of various institutional indicators on LCF, as well as ECOF for many countries and country groups, has been examined. In this framework, Lau et al. (2014) use the boundary test hypothesis to explore the relationship between institutional quality and carbon emissions and Malaysia’s economic development from 1984 to 2008. After the study, they concluded that when institutional quality characteristics and carbon emissions interact, quality institutions can lower carbon emissions and enhance environmental quality while promoting economic growth. The purpose of Abid (2016) research is to examine the effects of institutional, financial, and economic changes on CO2 emissions for 25 sub-Saharan African nations between 1996 and 2011. As a result of the study, he discovered that democracy and government stability suppressed carbon emissions in sub-Saharan African countries. Using time series data from 1971 to 2017, Sarkodie and Adams (2018) proved that political and institutional quality negatively predicts CO2 in South Africa. In their study, Salman et al. (2019) investigate the impact of institutional quality on the growth-emission link in a panel of three East Asian countries (Indonesia, South Korea, and Thailand) with data from 1990 to 2016. According to the panel cointegration test result, there is a positive and significant relationship between institutional quality and carbon emissions. Ali et al. (2019) concluded that institutional quality, including bureaucratic quality, legal system, and corruption control indicators, reduces CO2 emissions in 47 developing countries. For 47 emerging markets and emerging economies (EMDEs), which Le and Ozturk (2020) discussed, CADF and CIPS unit root tests were used in panel data between 1990 and 2014. The results of the study show that the quality of government spending in emerging market economies increases CO2 emissions through increased economic activities. Using data from 1984 to 2016, Hassan et al. (2020) employed an autoregressive distributed lag model (ARDL) for Pakistan. The study’s findings indicate that institutional quality in Pakistan contributes to rising CO2 emissions. Ni et al. (2022) examine the importance of institutional quality in improving the LCF of high resource-consuming economies with data from 1996 to 2019. Long-term results in the study prove that institutional quality improves LCF.

### Economic growth and LCF relationship

Two opposing theoretical views exist on the relationship between economic growth and ECOF (Panayotou 2003). The first theory states that economic growth reduces environmental quality by increasing ECOF (Bashir et al. 2020). In this context, Ahmed et al. (2019) analyzed the relationship between economic growth in Malaysia and ECOF using panel data for 1971–2014. The results are in the same direction as the first theory. In their study, Ahmed et al. (2022) examined the association between economic growth and ECOF for the G7 countries from 1985 to 2017. Empirical research points to the fact that economic growth raises ECOF. Akinsola et al. (2022) used data from 1983 to 2017 to study the effect of economic growth on ECOF in Brazil. Economic growth, according to the findings, promotes environmental damage. The second argument, on the other hand, contends that economic expansion significantly lowers ECOF (Hassan et al. 2019). The second theory argues that economic growth and ECOF have an inverse U-shaped connection (Destek and Sinha 2020). This connection predicts that trade and consumer trends will shift as the economy expands in favor of more environmentally friendly items. In this instance, products that cause less environmental harm will be produced, reducing ECOF and improving environmental quality (Borozan 2022). Aydin et al. (2023b) analyzed the relationship between economic growth and ECOF for 20 EU countries between 1990 and 2018. Empirical research has concluded that economic growth increases ECOF.

On the other hand, recent studies have focused on the relationship between economic growth and LCF. Some of these studies are from Liu et al. (2022), who used the ARDL bound test for Brazil with data between 1990 and 2018. They evaluated the impact of economic growth on the LCF. According to the results of the study, it has been proven that there is a long-term interrelationship between the selected indicators. Long-term resilience and economic growth deteriorate ecological quality. At the same time, economic expansion results in the LCF, which implies that it has the potential to forecast environmental quality over the long term. Awosusi et al. (2022b) used the data from 1980 to 2017 with the ARDL model for South Africa. Economic growth harms LCF. Akadiri et al. (2022) used the 1970–2017 annual data for India. The experimental results indicate a favorable correlation between economic growth and LCF.

## Data and model

This study investigated the validation of the LCC hypothesis in ten countries (Germany, Austria, Denmark, Finland, France, Netherlands, Spain, Italy, Sweden, and Switzerland) that invest in the highest environmental technology in the European Union. The description and sources of the variables used in this study are explained in Table 1. The LCC hypothesis is tested with four explanatory variables: environmental technologies, globalization, institutional quality, and economic growth. This relationship is modeled in Eq. 1.

1 $$\ln lc{f}_{it}={\beta}_0+{\beta}_1\ln gd{p}_{it}+{\beta}_2\ln gd{p}_{it}^2+{\beta}_3\ln e{t}_{it}+{\beta}_4\ln glo{b}_{it}+{\beta}_5i{q}_{it}+{\varepsilon}_{it}$$

**Table 1:** Variable definitions

Variables	Description	Source
Load capacity factor (lcf)	Biocapacity/ecological footprint (global hectares per person)	Global Footprint Network
Environmental technologies (et)	Patents on environment technologies (percentage)	OECD
Globalization (glob)	KOF (index)	Dreher, Axel (2006)
Institutional quality (iq)	ICRG Indicator of Quality of Government (index)	Knoema Database
Economic growth (gdp)	GDP per capita (constant 2015 US$)	World Development Indicator

where *ε*_*it*_ is the error term. We used logarithmic forms of all variables except institutional quality. The LCC hypothesis explains the U-shaped relationship between lngdp and lnlcf. If lngdp and lngdp2 are statistically significant and negative and positive, respectively, the LCC hypothesis is valid. Accordingly, lngdp decreases the lnlcf until the threshold value, increasing the lnlcf when this threshold value is exceeded.

## Methodology and empirical results

**Fig. 4:**
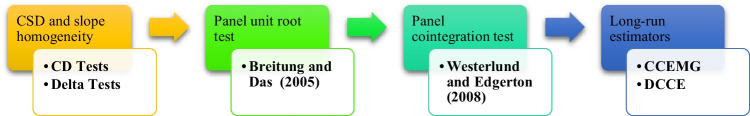
The steps of the empirical analysis. Source: authors

The empirical analysis part of this study was planned in four stages. These stages are presented in Fig. 4. Accordingly, the first step is the test of cross-sectional dependence (CSD) and slope homogeneity. Panel data produced by several cross-section units must take into account CSD. CSD has increased its importance for many time series with the effect of globalization. In this study, three different tests were used to test cross-section dependence. These tests are CDLM1, CDLM2, and CD, developed by Pesaran (2021) and Breusch and Pagan (1980). The homogeneity of Model 1 slopes is investigated using the Delta tests proposed by Pesaran and Yamagata (2008). Table 2 introduces these test results. According to the results, there is a CSD and heterogeneity of both variables and Model 1.

**Table 2:** Preliminary test results

Variables	CD_LM1_	CD_LM2_	CD
lnlcf	326.2330*	29.64456*	11.19457*
lngdp	1130.718*	114.4448*	33.35050*
lngdp2	1129.460*	114.3121*	33.32607*
lnet	876.9107*	87.69109*	29.08860*
lnglob	1292.048*	131.4504*	35.94209*
iq	478.5802*	45.70336*	16.93535*
Model	380.1094*	35.32363*	8.933454*
Slope homogeneity	Test statistics	*P*-value	
$\hat{\varDelta}$	7.815*	0.000	
${\hat{\varDelta}}_{adj}$	8.882*	0.000	

* denotes rejection of the null hypothesis at a 1% level of significance

We investigated the variable’s stationarity in the second step of the empirical analysis using the Breitung (2001)-Breitung and Das (2005) unit root test. This test allows cross-section dependence and has a good small sample property. The null and alternative hypotheses of this test are unit root and stationarity, respectively. Table 3 introduces the unit root test results. The results show that all variables are stationary at the first difference while they have a unit root at the level. In this case, the variables are I(1). This prior information is essential in selecting the cointegration test, which constitutes the third step of the analysis.

**Table 3:** Panel unit root test results

Variables	Level	First difference
lnlcf	−0.3681 (0.3564)	−2.3142 (0.0103)**
lngdp	0.8686 (0.8075)	−1.8149 (0.0348)**
lngdp2	0.8962 (0.8149)	−1.8696 (0.0308)**
lnet	−0.6297 (0.2645)	−3.4358 (0.003)*
lnglob	0.9071 (0.8178)	−2.1382 (0.0162)**
iq	−0.3787 (0.3524)	−1.4221 (0.0775)***

*, **, and *** denote rejection of the null hypothesis at 1%, 5%, and 10% levels of significance, respectively. Probabilities are in parentheses

We explore the long-run relationship between variables in Model 1 using Westerlund and Edgerton (2008) panel cointegration test. This test takes into account cross-section dependence and structural breaks. The cointegration model for this test is as follows.

2 $$\varDelta {\hat{S}}_{it}=\textrm{constant}+{\phi}_i{\hat{S}}_{it-1}+\sum_{j=1}^{pi}{\phi}_{ij}\varDelta {\hat{S}}_{it-j}+\textrm{error}$$

where ${\hat{S}}_{it}$ is a residual. The panel test statistics to test the null of no cointegration against the alternative hypothesis are defined as follows.

3 $$L{M}_{\phi }(i)=T{\hat{\phi}}_i\left(\frac{{\hat{\omega}}_i}{{\hat{\sigma}}_i}\right)\textrm{and}\ L{M}_{\tau }(i)=\frac{{\hat{\phi}}_i}{SE\left({\hat{\phi}}_i\right)}$$

where ${\hat{\phi}}_i$ and ${\hat{\sigma}}_i$ are the least square estimate and the estimated standard errors.

Panel cointegration test results are reported in Table 4. According to the cointegration test results, the load capacity factor and other explanatory variables move together in the long run. After this step, we estimated the long-run coefficients of Model 1 for the whole panel and each country. For this aim, we used two estimators: the common correlated effects mean group estimator (CCEMG) by Pesaran (2006) and the dynamic common correlated effects estimator (DCCE) by Chudik and Pesaran (2015). Panel estimation results are reported in Table 5.

**Table 4:** Panel cointegration test results

Tests	Test stat.	*P*-value
Westerlund and Edgerton (2008)		
tau_n	−2.469*	0.007
phi_n	−2.967*	0.002

* denotes rejection of the null hypothesis at a 1% level of significance

**Table 5:** Panel long-run estimation results

Variables	CCEMG		DCCE	
	Coeff.	*P*-value	Coeff.	*P*-value
lngdp	−35.440*	0.000	−53.654*	0.002
lngdp2	1.699*	0.000	2.484*	0.002
lnet	0.010	0.850	0.019	0.850
lnglob	**−**1.413***	0.061	**−**1.052	0.435
iq	0.021	0.953	**−**0.209	0.670

Bold values indicate robust results that are significant for both estimators

* and *** denote rejection of the null hypothesis at 1% and 10% levels of significance, respectively

The long-run coefficients of both estimators have validated the LCC hypothesis. On the other hand, the other explanatory variables have insignificant effects on the load capacity factor for both estimators. Lastly, we estimated each country’s long-run coefficients using the DCCE estimator. These results are reported in Table 6.

**Table 6:** Country-based long-run estimation results

Countries	lngdp	lngdp2	lnet	lnglob	iq
Austria	−18.2838	0.78292	0.215214*	−5.27879*	−2.68844*
Germany	−25.6547	1.141215	−0.28871	−0.02498	1.11414***
France	−120.449	5.729732	0.132487	0.465173	0.89860***
Italy	−501.033	24.07659	0.906414	−5.5227	−0.83149
Spain	−444.22**	21.49279**	−0.25112	−3.32529	0.620017
Denmark	17.4913	−0.67771	−0.04841	−3.33865	−1.63045
Finland	−33.5538	1.370919	−0.12113	−4.54479	−2.37424
Netherlands	−68.1796	3.238068	−0.05856	−2.10508	−4.54296
Sweden	−161.035	7.54184	0.723194	3.433393	−1.27074
Switzerland	−34.2695	1.432628	0.10979	−0.56306	0.007564

Bold values indicate robust results that are significant for both estimators

*, **, and *** denote rejection of the null hypothesis at 1%, 5%, and 10% levels of significance, respectively

The long-run coefficients can be explained as follows: firstly, the LCC hypothesis is valid only for Spain. Accordingly, lngdp decreases the lnlcf until the threshold value, increasing the lnlcf when this threshold value is exceeded in Spain. Secondly, environmental technologies increase the load capacity factor in Austria. For other countries, there is no significant relationship. Thirdly, globalization decreases the load capacity factor in Austria. Lastly, institutional quality reduces the load capacity factor in Austria while increasing it in Germany and France.

## Conclusion and policy recommendations

This study explores the impact of environmental technologies, institutional quality, globalization, and economic growth on LCF. The study selected ten countries (Germany, Austria, Denmark, Finland, France, Netherlands, Spain, Italy, Sweden, and Switzerland) that invest in the highest environmental technology in the EU. The data range of the study covers the years 1990 to 2019. According to both long-run estimators, the LCC hypothesis was found valid when the panel was evaluated. On the other hand, the LCC hypothesis is valid only for Spain. In this context, it has been determined that after a certain threshold for Spain, with the increase in economic growth, this country tends to have cleaner production technologies. With the widespread use of environmentally friendly production technologies, environmental pollution has decreased in the country. Good results have been obtained from the environmental protection regulations made in this country. Governments of other countries in the study must review their environmental measures to improve environmental quality.

Other study findings include environmental technologies for Austria increasing LCF. This finding is consistent with the investigations of Destek and Manga (2021), Hussain and Dogan (2021), Sharif et al. (2022), Ahmad et al. (2022), Awosusi et al. (2022b), Liu et al. (2022), Aydin et al. (2023a), and Apergis et al. (2023). The globalization variable reduces the LCF for Austria. The decrease in environmental quality with the increase of globalization is compatible with the results of the studies of Shahbaz et al. (2018) and Sharif et al. (2022). The institutional quality variable decreases LCF for Austria and increases it for Germany and France. These results are consistent with Salman et al. (2019), Le and Ozturk (2020), Hassan et al. (2020), Lau et al. (2014), Abid (2016), Sarkodie and Adams (2018), Ali et al. (2019), and Ni et al. (2022).

According to the results, it was determined that globalization decreased the LCF in Austria. In this framework, policymakers in the country in question can create environmental policies to measure the environmental viability of international investments or foreign direct investments. In addition, they can take some precautions against companies using old technologies. By offering special incentives to foreign investors, they can be encouraged to use cleaner technology that considers the environment. The national media may enhance environmental awareness, and the social contact network with other nations should be strengthened (Shahbaz et al. 2019). In other words, governments should consider globalization’s environmental consequences when designing their policies. In this context, governments may need to set emission standards. In this context, they can take various measures through the Emissions Trading System (ETS) and the Border Carbon Regulation Mechanism (CCRM). The EU has to prevent carbon leakage to make its climate policy more effective. As a result of this mechanism to be applied to imports, a source of income is created. Thus, third countries will be incentivized to harmonize green policies and reduce emissions. In addition to monitoring and reducing imports of goods with high carbon footprints, governments will develop carbon pricing policies to combat climate change. Developing policies in line with the World Trade Organization and other international systems will ensure compliance with ETS and CCRM rules in importing third-country origin products into the EU (European Commission (EC) 2021; Aydin and Degirmenci 2023). Companies that reduce environmental quality by ignoring these standards can be sanctioned. These sanctions can be carbon taxes, pollutant rights, and pollution credits. Such policies have the power to improve environmental quality as well as increase economic growth.

It was concluded that institutional quality decreased the LCF in Austria. Considering the impact of institutional quality on the LCF, attention needs to be paid to formal accountability mechanisms in regulatory bodies in this country. In this framework, it can be checked whether environmental regulations and commercial laws are applied. Administrative and governance values such as accountability, transparency, and government effectiveness can be added to institutional reforms by strengthening internal control systems (Le and Ozturk 2020). Adding these reforms will enable policymakers to enforce environmental protection laws and possibly make every citizen understand them. This way, institutions with relatively weak institutional quality will be replaced by institutions with solid institutional quality. Conversely, institutional quality increases the LCF in Germany and France. It shows that environmental regulations and trade laws are implemented in these two countries. These nations have six indicators—political stability, regulatory quality, government efficacy, the rule of law, accountability, and corruption control—contributing to higher environmental quality. Additionally, these nations have relatively low levels of behaviors, including lobbying, rent-seeking, bribery, and nepotism. The low level of these activities contributes to the countries’ decreased environmental pollution.

Climate change and environmental issues are of interest to the EU. As a result, the EU strongly committed to environmental sustainability in 2016 by signing the Paris Climate Agreement. It is now required to eliminate carbon and several other harmful pollutants due to the challenges brought on by climate change due to global warming (United Nations n.d.). Two major environmental initiatives will be implemented in 2019 with the European Green Deal, which has objectives nearly identical to those of the Paris Climate Agreement. By 2030, greenhouse gas emissions must be reduced by at least 55%, according to the first environmental policy. Developing and diffusing environmental technologies for the EU can be crucial in lowering greenhouse gas emissions. The study’s findings support that environmental technology improves Austria’s environmental quality. In this context, 242 environmental patents in Austria in 2019 support this role (OECD 2023). Another environmental policy is making the European continent the first climate-neutral zone by 2050. The proposed “Fit for 55” green package, which contains several legal measures, was submitted to the commission in 2021 to meet the environmental objectives that the EU had decided upon in this respect.

The UN and the EU adopted the Sustainable Development Goals (SDGs) to preserve environmental quality. SDG-7 aspires to make clean energy research and technologies, such as energy efficiency, renewable energy, and cleaner fossil fuel technology, more widely accessible by 2030. In this situation, greater international collaboration and encouragement of investment in clean energy technology sectors are required to attain this goal. Furthermore, SDG-9 seeks to enhance environmental quality by promoting cleaner and more environmentally friendly technology by 2003, per each nation’s capacity (SDG Report, 2023). In the study, the increase in Austria’s investments in cleaner environmental technologies positively affected LCF. A value greater than 1 for LCF contributes to SDG targets and positively affects environmental quality. In addition to these countries, the use of environmentally friendly technologies is increasing with strong public support, especially in the European region. Environmental quality will rise as environmentally friendly technology expands and develops. Finally, the EU indirectly promotes investments in environmentally friendly technology to accomplish the aims of the European Green Deal with plans and programs like Horizon Europe and Next, Generation EU in conjunction with the Paris Climate Agreement and the “Fit for 55” projects. Additionally, institutions and researchers should be encouraged by policymakers to employ eco-friendly technologies. These incentives can take the form of tax exemptions and subsidies. In line with the results obtained, policymakers should focus on improving the environmental impact of environmental technologies to promote and sustain environmental sustainability. Given the accelerating pace of environmental issues such as overcrowding, overconsumption, climate change, and new global markets, investing in these environmental technologies has become imperative. Therefore, countries need to reconsider all kinds of technological investment policies. Integrating environmental policies will encourage the creation of more informed technology structures that control risks and uncertainties with emerging developments in environmental technology.

This study can be a guiding study for future studies on this subject. The effects of these variables on LCF may be the subject of research, both by using different methodological techniques and for distinct nations and nation groups in the future. The study was also restricted to 2019 due to data availability. Future studies may analyze this issue using extended data. In the literature, institutional quality and globalization can be used to affect environmental quality. However, there is a small database of patents related to the content of environmental technologies. Since he can be a researcher in this field, the environmental technologies and environmental quality literature can be expanded for future studies.

## Data Availability

The data is accessible from the corresponding author upon request.
